# Phototrophs in Unique Habitats of Thermomineral Springs in Central Serbia

**DOI:** 10.3390/life15020169

**Published:** 2025-01-24

**Authors:** Ana Milićević, Slađana Popović, Vanja Milovanović, Vesna Karadžić, Željko Savković, Vukašin Bjelica, Jelena Krizmanić, Gordana Subakov-Simić, Olga Jakovljević

**Affiliations:** 1 Department of Algology and Mycology, Institute of Botany and Botanical Garden “Jevremovac”, Faculty of Biology, University of Belgrade, Takovska 43, 11000 Belgrade, Serbia; 2 Institute of Public Health of Serbia Dr Milan Jovanović Batut, dr Subotića 5, 11000 Belgrade, Serbia; 3 Institute of Zoology, Faculty of Biology, University of Belgrade, Studentski Trg 16, 11000 Belgrade, Serbia

**Keywords:** cyanobacteria, eukaryotic algae, extreme environments

## Abstract

Thermomineral springs are unique aquatic habitats characterized by high temperatures or mineral-rich water and often host specialized microbial communities. In Serbia, these springs represent an important but under-researched ecological resource whose diverse physicochemical properties are shaped by their geological context. In this study, the physical and chemical properties of Serbian thermomineral springs and their relationship with phototrophic communities in different substrates are investigated. Phototrophic biofilms were categorized into fully submerged and splash zone biofilms, with the former showing higher primary production. Cyanobacteria, Chlorophyta, and Bacillariophyta were recorded, with Bacillariophyta being the predominant division in terms of diversity, followed by Cyanobacteria. Among Cyanobacteria, coccoid forms like *Aphanocapsa*, *Chroococcus*, *Gloeocapsa* and *Synechococcus* dominated splash zones, while trichal forms such as *Leptolyngbya*, *Oscillatoria* and *Pseudanabaena* were abundant in submerged biofilms, forming thick mats. Unique cyanobacterial taxa, including *Desertifilum*, *Elainella*, *Geitlerinema*, *Nodosilinea* and *Wilmottia*, were identified through molecular analysis, underscoring the springs’ potential as habitats for specialized phototrophs. Diatom communities, dominated by *Nitzschia* and *Navicula*, exhibited site-specific species influenced by microenvironmental parameters. Statistical analysis revealed ammonia, total nitrogen, and organic carbon as key factors shaping community composition. This study enhances the understanding of these ecosystems, emphasizing their conservation importance and potential for biotechnological applications.

## 1. Introduction

Hot and thermal springs occur in many parts of the world and are defined by the temperature of the water [1]. Thermomineral springs represent special habitats and can play an important role in ecosystems from an ecological perspective [2]. They represent niches with specific physical and chemical conditions (e.g., high temperatures, specific pH, and high conductivity) [3], in which high concentrations of soluble cations (such as sodium, calcium, silicon, or potassium) and anions (chlorides and sulfates) stand out [4]. These habitats can harbor rare, endemic, and endangered species and even relict taxa, especially when these ecosystems are isolated from others [3,5].

Microorganisms, including Cyanobacteria and eucaryotic algae, that utilize light for energy are found in a variety of habitats ranging from common ecosystems to harsh habitats with extremes of temperature, pressure, salinity, pH, and other environmental factors [6]. These habitats can even include aerosols, where phototrophic microorganisms such as *Trentepohlia* spp. thrive on surfaces such as building walls and tree bark [7]. In hot and cold deserts, Cyanobacteria and eucaryotic algae form microbial mats that contribute to carbon fixation [8]. Acidic and saline lakes, such as the Dead Sea, host specialized phototrophs which tolerate extreme pH and salinity [9,10]. Of all phototrophs, Cyanobacteria are most known for their exceptional adaptability that enables them to inhabit a variety of extreme environments. For example, they utilize unique physiological mechanisms such as high levels of carotenoids and other protective pigments to mitigate oxidative stress and damage from UV radiation. They can also form specialized cells such as akinetes and heterocysts to survive prolonged desiccation or nutrient scarcity, which is particularly advantageous in deserts or polar habitats [10,11]. Their ability to withstand these extreme conditions makes them valuable models for understanding life in extraterrestrial environments. Phototrophic microorganisms are also commonly found at the place of the emergence of thermomineral springs, where they play a key role in these ecosystems, particularly in relation to the cycling of minerals and organic matter. Among phototrophic microorganisms, Cyanobacteria are frequent inhabitants of such places, where they are very well adapted to the extreme habitat conditions and dominate as primary producers. Some scientists believe that the ancestors of Cyanobacteria were probably the oldest primary producers and used thermal springs as a refuge [12,13,14]. Studies on the microbial communities of thermomineral springs have long been focused on prokaryotic organisms, while eukaryotes remained poorly studied [15]. Among eukaryotes, diatoms represent a large group of microalgae that can be found in almost all ecological niches [16]. Thanks to a series of physiological adaptations and different mechanisms, they can be found in extreme habitats, including hot springs and highly mineralized waters [17]. Diatoms can adjust their membrane lipid composition to maintain fluidity at high temperatures to ensure proper cell function [18]. They are also able to tolerate high concentrations of minerals such as silica through efficient silicification processes that increase the stability of their frustules, the silica-based cell walls [19]. These adaptive strategies allow diatoms to colonize extreme niches that are inhospitable to many other organisms, making them an important component of these aquatic ecosystems. In addition to Cyanobacteria and diatoms, green algae should also be pointed out. It is a group of algae that inhabit aquatic and aerophytic habitats, but also many extreme habitats, so they are important in the research of thermomineral springs [20].

Thermomineral springs are studied from different aspects and microbiological studies provide a better understanding of the habitat, their extremophilic microorganisms, and their adaptations [21]. As there are a large number of different thermal and thermomineral springs in Serbia, these localities have already been studied and are mentioned in several previous and recent publications [22,23,24,25,26]. However, there are still many that are unexplored, and there are many novel methods that can be applied for research purposes but have not yet been used. The aim of this study was to investigate the phototrophic community developing at the emergence of five thermomineral springs in Serbia. In this study, cyanobacterial and algal taxa associated with selected thermomineral springs were investigated in detail. Primary production, environmental, and water parameters were evaluated and, where possible, correlated with the phototrophic component. In addition, different types of biofilms associated with phototrophs were investigated. For the first time, cyanobacterial taxa were cultivated and analyzed using molecular methods. This study not only contributes to the understanding of phototrophic microbial communities in thermomineral springs but also lays the foundation for future research. Comparative studies with microbial communities from other extreme environments, as well as hot springs from different geographical regions or chemically different thermal waters, could further elucidate the adaptive mechanisms and evolutionary relationships. These approaches will expand knowledge of how Cyanobacteria, diatoms, and other algae thrive under extreme conditions and their wider ecological significance.

## 2. Materials and Methods

### 2.1. Study Area

**Figure 1:**
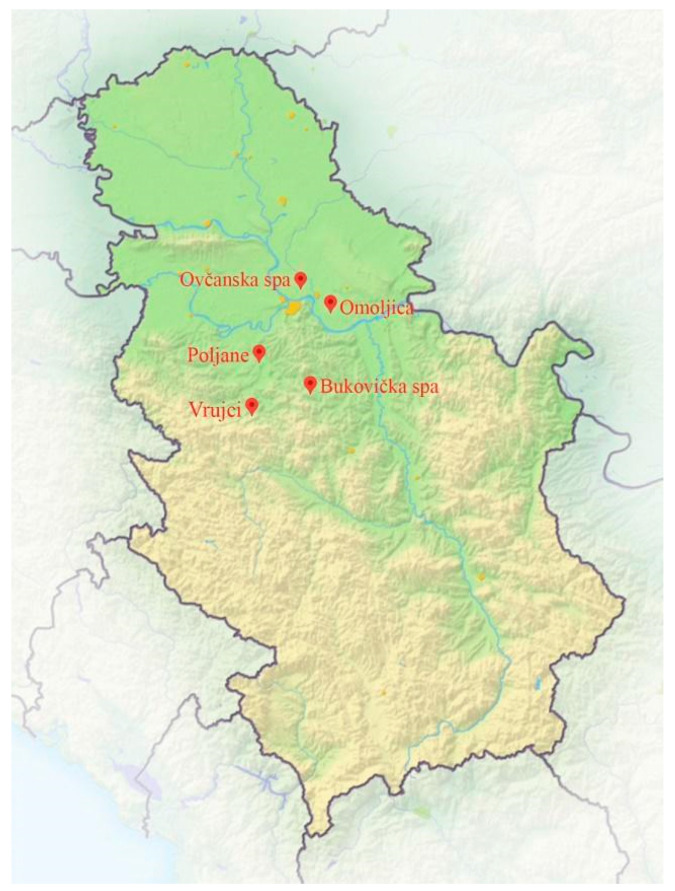
Location of thermomineral springs in Serbia (map source: https://www.alo.rs/data/images/2015-11-19/21475_profimedia-0088349174-1000x0_orig.jpg (accessed on 23 November 2024)).

The study area included five thermomineral springs in central Serbia (Figure 1), of which Omoljica, Ovčanska spa, Vrujci (only one site), and Poljane were natural thermomineral springs, while taps were present in Vrujci (two sites) and Bukovička spa. According to Risler, thermomineral springs are those that meet at least one of the following two criteria: (i) thermality, i.e., springs whose water temperature at the outlet is higher than the average annual air temperature, or (ii) mineralization, i.e., whose total mineralization is between 1 and 6 g/L [27]. All springs sampled in this study met one or both of these criteria, and their water properties are presented in the Results Section.

### 2.2. Analysis of Ecological and Water Parameters

Sampling was carried out between November and December 2023, visiting the aforementioned thermomineral springs to sample the water, the biofilms that develop at place of emergence or water splashing, and the sediment and mud (if present), and to measure the necessary ecological parameters. Physical and chemical parameters of the water (temperature (T), pH, total dissolved solids in water (TDS), electrical conductivity (EC), and oxygen concentration (O_2_)) were measured in situ using digital field instruments made by Eutech Instruments Oakton® and YSI ProODO Optical Dissolved Oxygen Meter. Regarding the number of measurements, all parameters measured in situ were measured three times and the mean value is shown in Result section. In addition, immediately after the field work, the water samples were sent to the Institute of Public Health “Milan Jovanović Batut”, where they were further analyzed according to the standard analytical procedures. In addition to the chemical and physical parameters of the thermomineral water, we measured the humidity and temperature of the biofilm that develops at the place of water emergence or water splashing, as well as the distance of the biofilm from the water source (if the biofilm was not at the source itself), and determined the color of the biofilm and its exposure.

### 2.3. Sampling Sites and Sampling Procedure

**Figure 2:**
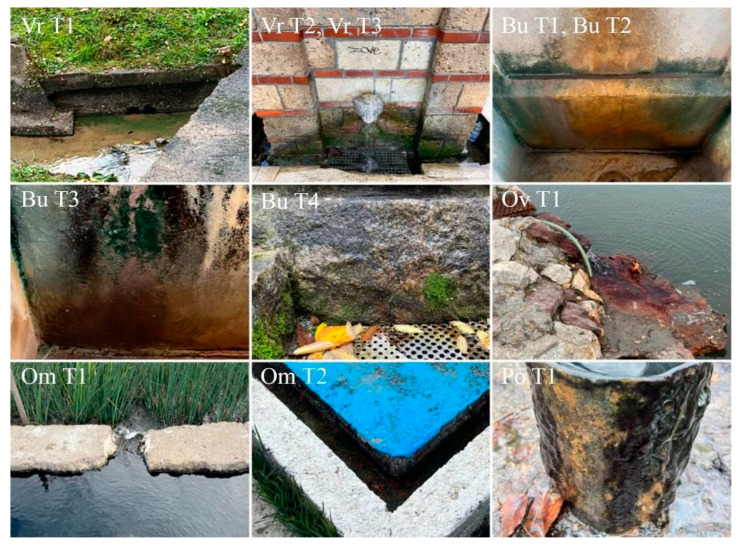
Biofilm sampling sites for microbiological analyses (Vr T1, Vr T2—Vrujci; Bu T1, Bu T2, Bu T3, Bu T4—Bukovička spa; Ov T1—Ovčanska spa; Om T1, Om T2—Omoljica; Po T1—Poljane).

Considering all five thermomineral springs mentioned (Table 1), a total of 14 sampling sites were selected: 11 samples of biofilms-fully submerged or in the splash zone (in all five springs) (Figure 2) and three samples of sediments (Omoljica, Ovčanska spa, and Vrujci). From two thermomineral springs, we obtained the mud used for commercial purposes (Bukovička spa and Vrujci). Only sites with biofilms are shown in Figure 2. Samples were collected by scraping with a sterilized scalpel and spatulas (biofilms) and placing the material in sterile plastic bottles from the predominant substrate at each site (e.g., rocks, concrete walls of the spring, and sediments). Where possible, sediments were also collected in the same way as biofilms. The mud, which had previously been prepared for therapeutic purposes, was obtained ready-made from the therapist. The sampling process is carefully performed to avoid contamination or disturbance of the surrounding environment. The collected samples were transported to the laboratory in sterile plastic bags. The diatom samples were preserved in 4% formaldehyde before further processing in the laboratory. Samples were collected not only for phototrophic analysis, cultivation, and molecular analysis, but also for chlorophyll *a* analysis. A circular plastic template was used to delineate sections on substrates from which biofilm samples were taken for the extraction of chlorophyll *a* [28]. The surfaces on which the template was applied were relatively smooth and showed only minor irregularities.

### 2.4. Analysis of Phototrophic Microorganisms

The preparation of the temporary slides of Cyanobacteria removed from the biofilm was carried out according to the method of Popović et al. [28]. A portion of the biofilm material was mixed with a drop of glycerol on a glass slide and observed directly under a Zeiss Axio-Imager M.1 light microscope with AxioVision 4.9 software.

A detailed analysis of the diatoms required special laboratory preparation of the samples and making permanent slides. The removal of the organic content of the diatoms was carried out according to the method of Taylor et al. using HCl and KMnO_4_ [29]. The samples obtained were used to make permanent slides by immersing the dried cell walls in the synthetic medium Naphrax^®^.

After preparing slides of eucaryotic algae and Cyanobacteria and observing them under the microscope, the taxa were identified using identification keys [30,31,32,33,34]. Following the qualitative analysis, a quantitative analysis was carried out within the diatoms community. It is presented in the form of the percentage representation of each taxon in the sample by counting 400 valves on each slide [35].

### 2.5. Analysis of Chlorophyll a/Primary Production

Chlorophyll *a* was extracted from biofilm samples following a modified protocol based on Popović et al. [36]. Upon collection, samples were stored in sterile polyethylene bags and processed immediately upon arrival at the laboratory. The biofilm samples were weighed and placed in 20 mL of hot 100% ethanol for pigment extraction. After homogenization, the extracts were filtered, and the absorbance of the filtrate was measured using a spectrophotometer (Cecil CE 2501) at wavelengths of 665 nm (specific to chlorophyll *a*) and 750 nm. The measurements at same wavelengths were repeated after acidification of the filtrate to address degradation products and ensure accurate quantification. The chlorophyll *a* concentration was calculated using the modified standard formula µg/cm^2^ = (A − Aa)/Kc × R/(R − 1) × (10^3^Ve)/(Vsd) (where A = A_665_–A_750_ refers to the absorbance of extract before acidification; Aa = A_665_–A_750_—absorbance of extract after acidification; Ve—volume of the extract (mL); vs.—volume of the filtered sample (L), in our case, weight of the sample (kg or g); Kc = 82 L/µg cm—specific operational spectral absorption coefficient for chlorophyll *a*; R = A/Aa—ratio A/Aa for a solution of pure chlorophyll *a* which is transferred to phaeophytin by acidification; d—the path length of the optical cell (cm); 10^3^—dimensional factor to fit Ve). This approach focuses specifically on chlorophyll *a*, reducing potential interference from other chlorophyll types and degradation products.

### 2.6. Cultivation of Cyanobacteria

Cyanobacteria were cultured in sterile plates with BG11 medium with 1.3–1.5% agar, prepared by autoclaving at 114 °C for 25 min and adjusting the pH to ~7.5 [37,38]. After slight cooling, the medium was poured into sterile Petri dishes. Biofilm samples were inoculated, and dominant taxa were allowed to develop. Cultures were subcultured every 3 weeks from February to October, examined microscopically, and subjected to molecular analyses.

### 2.7. Molecular Analyses

For PCR amplification of the small subunit ribosomal RNA (16S rRNA) gene, cyanobacterial DNA was extracted using the Quick-DNA™ Fungal/Bacterial Miniprep Kit (Zymo Research, Irvine, CA, USA). The extracted DNA was used as a template for PCR reactions with primers specifically designed for cyanobacterial 16S rRNA gene regions. The forward primer CYA359F (5′-GGGGAATYTTCCGCAATGGG-3′) and the reverse primer CYA781R (5′-GACTACTGGGGTATCTAATCCCWT T-3′) were used to amplify a ~600 bp fragment of the gene. Each 25 µL PCR reaction contained 1 μL of template DNA, 1 μL of each primer, 12.5 μL 2× PCR TaqNova-RED (Blirt), and 9.5 μL of deionized water. PCR was performed in a thermal cycler under the following conditions: initial denaturation at 95 °C for 2 h, followed by 32 cycles of 95 °C for 30 s, 52 °C for 2 min, and 72 °C for 2 min with a final extension at 72 °C for 10 min. The amplified DNA fragments were fractionated in 1% agarose gels in 0.5× TBE buffer and visualized by MIDORIGreen staining (NIPPON Genetics EUROPE, Düren, Germany) under UV illumination. The resulting PCR products were sent for sequencing (Macrogene, The Netherlands). For primary identification, the sequences were compared with other related sequences from the NCBI database using the BLAST tool (BLAST+ 2.7.1 from NCBI). A phylogenetic tree was made using MEGA11 software. The alignment of sequences was performed using ClustalW algorithm and the phylogenetic tree was built using the neighbor joining phylogeny model (1000 bootstrap replicas). The Kimura 2 parameter model was determined as the best for estimating genetic distances between tested sequences. *Escherichia coli* strain 23 JX467700.1 was used as an outgroup.

### 2.8. Statistical Analysis

Principal component analysis (PCA) was used to represent the documented phototrophic microorganisms sorted into appropriate groups (coccoid Cyanobacteria, simple trichal Cyanobacteria, Chlorophyta, and Bacillariophyta) in relation to the moisture content of the biofilm and the sample type at the place of the emergence of thermomineral waters. The phototrophic microorganisms were sorted into appropriate groups using the “trait average” option available in the software. Considering the moisture content, the biofilms were categorized into six groups: G2 (10–20%), G3 (20–30%), G4 (30–40%), G9 (80–90%), G10 (90–100%), and SB (fully submerged biofilms). Considering the sample type, sediment and two types of biofilms (those that are water-splashed and those that are completely submerged or constantly moistened) were included in the analysis. Redundancy analysis (RDA) was used to relate the documented cyanobacterial and algal taxa to physical and chemical water parameters. Significant variables were assessed using the “interactive forward selection” option. Analyses were performed using Canoco 5 software [39].

## 3. Results

### 3.1. Physical and Chemical Parameters of Thermomineral Springs

The results of the physical and chemical water parameters are shown in Table 2. The water temperature varied considerably between springs, ranging from 8.5 °C in Bukovička spa–Knjaz Mihailo (lowest) to 25.1 °C in Vrujci (highest), indicating different thermal conditions. Bukovička spa–Topli izvor had the highest turbidity (9.46 NTU), while Vrujci and Poljane had the lowest values of this parameter, indicating clearer water. The pH ranged from 6.86 (Bukovička spa) to 8.19 (Poljane). Total dissolved solids (TDS) and electrical conductivity (EC) were highest in the Ovčanska spa, indicating a higher mineral content, especially when compared to some other springs such as the Bukovička–Knjaz Mihailo spring. Oxygen saturation varied greatly, with Bukovička–Knjaz Mihailo spring having the highest oxygen content (92.5%) and Poljane the lowest (17.6%). The ammonia nitrogen concentration reached its highest value in the Ovčanska spa (19 mg/L), while the nitrite-N content was below the detection limit in all springs. The Bukovička (Topli izvor) spa stood out with its fluoride, silicate, and bicarbonate concentrations. High bicarbonate concentrations were also recorded in Omoljica. Calcium and magnesium concentrations vary, with the highest values found in Vrujci (Ca^2+^) and Ovčanska spa (Mg^2+^).

### 3.2. Sampling Sites Characteristics

As depicted in Figure 2, different colorations of the biofilms were observed. In addition, different types of biofilms were encountered during sampling (Figure 2). Three types of biofilms could be distinguished: fully submerged biofilms (Vr T1, Ov T1, Om T1, Om T2, and Po T1) and biofilms that were splashed by thermomineral water and developed on the walls of the taps (Vr T2, Vr T3, Bu T1, Bu T2, Bu T3, and Bu T4). Biofilm moisture was measured for all biofilms that were in any way outside the water. In addition, biofilms were differentiated according to their thickness, where well-developed biofilms (i.e., Po T1) and less developed biofilms (mainly epilithic, on the walls of the taps, i.e., Bu T1, Bu T2, Bu T3, and Bu T4) were observed.

### 3.3. Results on Analysis of Phototrophic Microorganisms

The samples examined showed the presence of three groups of phototrophic microorganisms: Cyanobacteria, Chlorophyta (green algae), and Bacillariophyta (diatoms) (Table 3). Looking at the number of taxa, the Bacillariophyta were the predominant division, followed by Cyanobacteria. Of the Chlorophyta, only two taxa were recorded.

Most of the Cyanobacteria were filamentous forms from the simple trichal group, of which genera *Leptolyngbya* and *Phormidium* had the highest number of recorded species, followed by *Oscillatoria* and *Pseudanabaena*. In the coccoid group, eight genera were identified, of which genera *Gloeocapsa* and *Synechococcus* were the most diverse, but *Aphanocapsa* and *Chroococcus* are also worth mentioning. Not a single heterocytous Cyanobacterium was detected. The largest number of cyanobacterial taxa was found in samples from Bukovička, Vrujci, and Omoljica. Many taxa were found at only one sampling site, such as *Aphanothece saxicola* Nägeli and *Chroococcus thermalis* (Meneghini) Nägeli in Vrujci or *Jaaginema geminatum* (Schwabe ex Gomont) Anagnostidis and Komárek and *Pseudanabaena thermalis* Anagnostidis in Omoljica, while some taxa were detected at more than one site, such as *Leptolyngbya boryana* Anagnostidis and Komárek (three sites), *Chroococcus varius* A.Braun (two sites), and *Gloeocapsa atrata* Kützing (two sites) (Table 3).

The most diatom taxon-rich genera were *Nitzschia* and *Navicula*, respectively. The samples from the Vrujci show the highest diatom diversity and those from the Bukovička spa the lowest. The natural spring in Vrujci differs significantly from the samples from the tap wall in terms of the number and composition of species—almost twice as many taxa have been identified in the natural thermomineral spring than at the tap. A large number of *Nitzschia palea* (Kützing) W.Smith was found in the Omoljica thermomineral spring. *Nitzschia thermaloides* Hustedt was found in the Ovčanska spa, but also in Omoljica and Vrujci. *Adlafia muralis* (Grunow) Monnier and Ector and *Planothidium frequentissimum* (Lange-Bertalot) Lange-Bertalot were only found in biofilm collected at the emergence of the thermomineral spring in Poljane (Table 3).

### 3.4. Chlorophyll a

**Figure 3:**
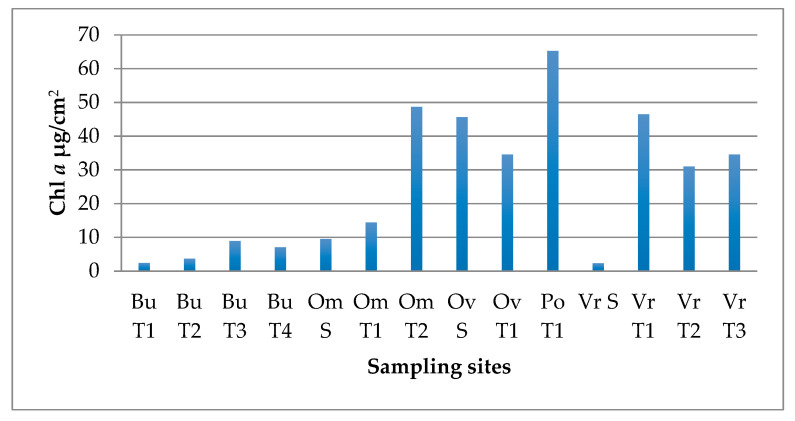
The concentration of chlorophyll *a* (Chl *a*) expressed as µg/cm^2^. The unit μg/cm^2^ tells us how much chlorophyll *a* has been extracted from a biofilm that is sampled from a substrate surface of one square centimeter.

The chlorophyll *a* (Chl *a*) content varied between the different sampling sites (Figure 3). The mud samples from Bukovička and Vrujci spa had barely detectable Chl *a* levels, all below 1 µg/cm^2^ Chl *a*, and were not shown in Figure 3. Lower Chl *a* values were measured in biofilms from sites in the Bukovicka spa, at two sampling sites in Omoljica (Om T1 and Om S), and at one site in Vrujci (Vr S). Higher concentrations were measured in many samples from Vrujci, Ovčanska spa and one site in Omoljica (Om T2), all above 30 µg/cm^2^ Chl *a*. Poljane stood out, and Chl *a* extracted from the phototrophic biofilm reached values over 60 µg/cm^2^ Chl *a*.

### 3.5. Molecular Analysis of Cultured Cyanobacteria

**Figure 4:**
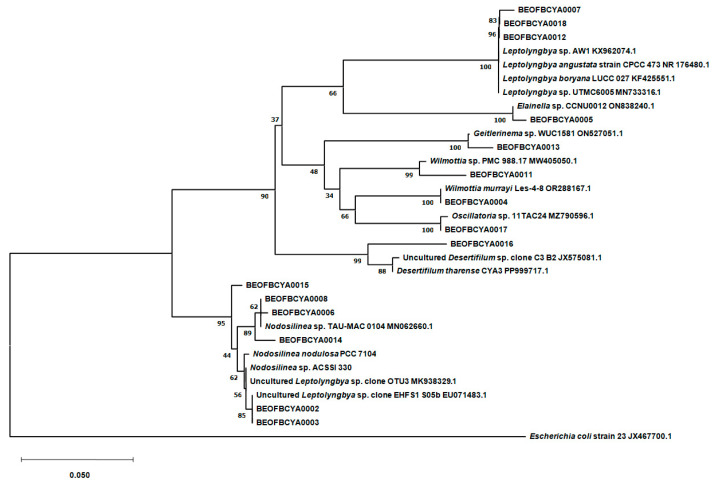
Neighbor joining phylogenetic tree based on 16S rRNA gene sequences showing the relationship between cyanobacterial strains isolated from thermomineral springs in Serbia and closely related reference sequences.

A total of 15 taxa were identified from cultures using molecular methods (Figure 4). Isolates BEOFBCYA0007, BEOFBCYA0012, and BEOFBCYA0018 clustered together with *Leptolyngbya* species in a well-supported clade. Likewise, BEOFBCYA0005 clustered together with *Elaniella* sp. Isolates BEOFBCYA0004, BEOFBCYA0011, and BEOFBCYA0017 clustered together with *Wilmottia* species. Although BEOFBCYA0017 showed the greatest homology with *Oscillatoria* sp., it is probable that this isolate is actually a member of the *Wilmottia* genus due to the clustering results and morphological characteristics. Additionally, isolate BEOFBCYA0016 clustered together with *Desertifilum* species in a well-supported clade. Finally, BEOFBCYA0002, BEOFBCYA0003, BEOFBCYA0006, BEOFBCYA0008, BEOFBCYA0014, and BEOFBCYA0015 isolates clustered together with *Nodosilinea/Leptolyngbya* species in a separate clade. In this case, morphological and molecular data suggest that all the isolates are members of the genus *Nodosilinea*.

### 3.6. Statistical Analyses

**Figure 5:**
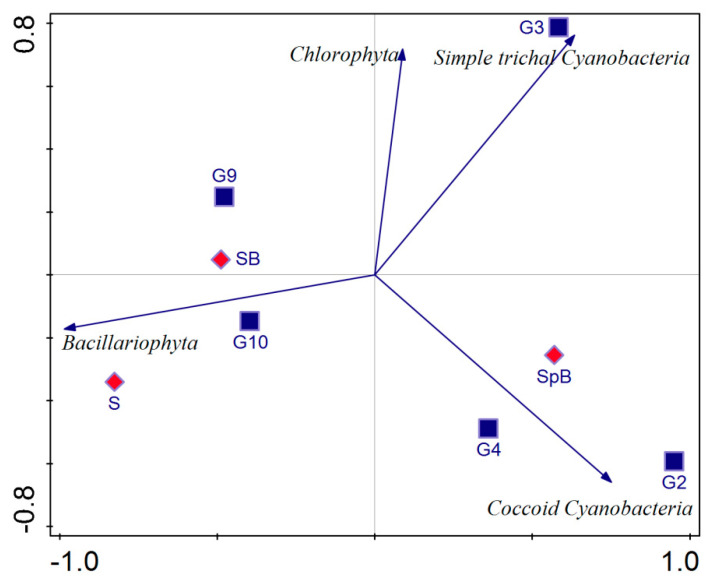
PCA showing phototrophic microorganisms in relation to the moisture content of the biofilm (G2 (10–20%), G3 (20–30%), G4 (30–40%), G9 (80–90%), and G10 (90–100%) and SB (fully submerged biofilms)) and the sample type and type of biofilm (sediment (S), water-splashed biofilms (SpB), and fully submerged biofilms (SB)). The blue arrows represent the groups of phototrophic microorganisms, the blue squares correspond to the moisture content of the biofilm, and rhombs represent the type of sample and the type of biofilm.

The documented phototrophic microorganisms sorted in appropriate groups (coccoid Cyanobacteria, simple trichal Cyanobacteria, Chlorophyta, and Bacillariophyta) were related to the moisture content of the biofilm and the sample type—sediment and two types of biofilm (fully submerged and water-splashed biofilm) using PCA (Figure 5). All variables account for 68.17% of the total variation. The first axis accounts for 62.33% of the variation, while the first and second axes together explain 96.15%. Taking these biofilm characteristics into account, two phototrophic groups could be clearly distinguished. Coccoid Cyanobacteria correlate positively with biofilms that have developed at sites that are splashed by water, i.e., biofilms with lower moisture content (G2–G4). On the other hand, Bacillariophyta dominate in sediment and submerged biofilms or in those characterized by higher moisture content (G9 and G10).

**Figure 6:**
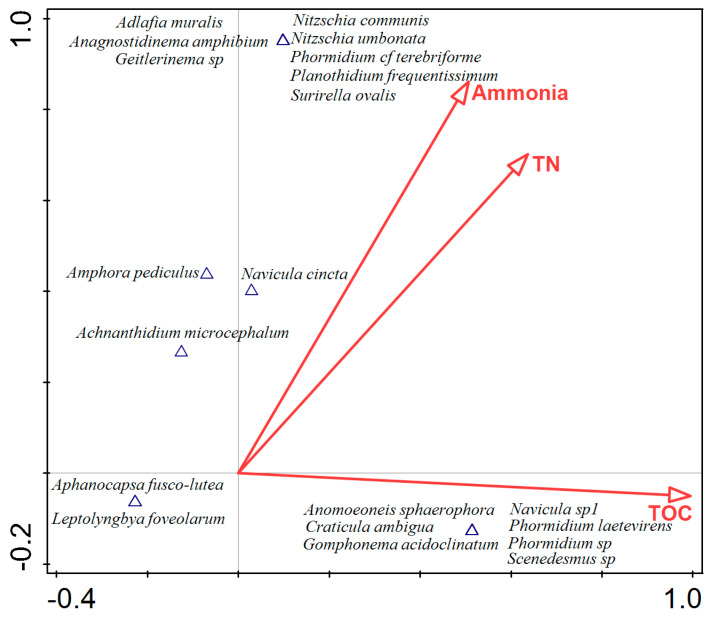
RDA showing the relationships between the 20 best fitted Cyanobacteria and algal taxa and the significant physical and chemical water parameters (ammonia; TN—total nitrogen; TOC—total organic carbon). The significant environmental parameters are represented with red arrows, while the triangles (▲) represent phototrophic taxa. The cyanobacterial and algal taxa are plotted according to their correspondence with the environmental variables.

The RDA ordination diagram shows 20 of the best-fitted taxa in relation to three significant factors—ammonia, TN, and TOC, selected by using the “Interactive forward selection” option (Figure 6). All variables account for 49.31% of the total variation. Ammonia explains 17.3%, TOC 17.7%, and TN 14.3% of the total variation. The variation explained by the first two axes (cumulative) is 35%, of which the first axis explains 17.73%. As can be seen in the ordination diagram, each of these variables corresponds positively with a certain group of taxa: ammonia and TN with the group of taxa in the upper part of the ordination diagram (representatives of *Adlafia, Anagnostidinema, Geitlerinema, Nitzschia*, *Phormidium, Planothidium* and *Surirella*), and TOC with the group of taxa in the lower part of the ordination diagram (representatives of *Anomoeoneis, Craticula, Gomphonema, Navicula, Phormidium* and *Scenedesmus*).

## 4. Discussion

Thermomineral springs are characterized by relatively stable environmental conditions in which the temperature and chemical parameters generally show only minimal seasonal fluctuations [24]. The data on the physical and chemical parameters of the water indicate considerable differences between the individual sites, reflecting the specificity of each thermomineral spring (Table 2). The water temperature in the explored thermomineral springs ranged from 8.5 °C in Bukovička spa–Knjaz Mihailo to 25.1 °C in Vrujci. Higher temperatures such as those in Vrujci (25.1 °C) are consistent with those in Karlovy Vary, where thermal springs are typically warmer than 20 °C and host a diverse community of thermophilic microorganisms [40]. These higher temperatures favor the growth of thermophilic organisms, especially filamentous Cyanobacteria such as *Leptolyngbya* and *Phormidium*, which have already been detected in geothermal springs in Iceland and Turkey [41]. Similarly, Šaraba et al. highlighted that the presence of *Leptolyngbya* and *Phormidium* in Serbian thermomineral springs indicates their adaptability to these stable yet extreme environments, supporting their dominance in biofilms at multiple localities [24]. The species of these genera thrive due to their ability to withstand high temperatures and nutrient limitations [42]. A prevalence of *Leptolyngbya* has also been detected in studies on Spanish springs, further supporting the idea of a common ecological strategy of Cyanobacteria in thermomineral habitats [43]. In addition to temperature, Serbian springs also exhibit significant variations in turbidity and pH. For example, the Bukovička spa–Topli izvor spring has the highest turbidity (9.46 NTU) and a pH of 6.86, while the Poljane spring is the clearest (0.02 NTU) and has the highest pH (8.19). These fluctuations are influenced by the natural mineral content, as observed in Berchtesgaden [44]. The lower pH in the Bukovička spa indicates stronger mineralization, similar to the acidic, sulfate-rich waters in Karlovy Vary (Czech Republic) [40]. TDS, EC, and mineral content, especially ammonia and calcium, varied greatly between the springs in Serbia. The Ovčanska spa showed the highest TDS and EC values, indicating higher mineralization compared to springs such as Bukovička–Knjaz Mihailo. This trend is consistent with European springs, such as those in Italy and Spain, where local geology influences ion composition [45]. For anions, bicarbonates dominated in Bukovička Spa–Topli izvor (3665 mg/L) and Omoljica (1202 mg/L), which is consistent with results from Greek thermal springs, where bicarbonate-rich water is associated with carbonate rocks [46]. Chlorides peaked in Vrujci (995 mg/L), possibly related to deep hydrothermal circulation, similar to the Maragheh springs with high chloride levels due to geochemical processes [47]. Sulfates were generally low in the Serbian springs, with the Bukovička–Knjaz Mihailo spa having modest concentrations (42.2 mg/L), in contrast to the higher sulfate levels in the Atlantic springs, which were influenced by the oxidation of sulfide minerals [43]. Cation concentrations were also highly variable. Calcium was highest in Vrujci (75.3 mg/L), suggesting interactions with calcium-rich minerals, a pattern also observed in the Maragheh springs [47]. Magnesium reached a maximum value in Ovčanska spa (86.5 mg/L). Fluoride (5 mg/L) and silicates (81.59 mg/L) were particularly prominent in Bukovička Spa–Topli izvor, reflecting its unique geochemistry. These elevated silicate values are similar to those found in Greek springs with travertine deposits, where silicates play a crucial role in biomineralization processes [46].

A clear separation of biofilms (all shown in Figure 2) according to the conditions under which they develop is important, as different microconditions are formed that can significantly influence the composition and structure of biofilms. Biofilms that formed underwater and were fully submerged mostly exhibited well-developed structures, probably due to the constant presence of mineral-rich water, that promotes the development of abundant microbial communities adapted to the given conditions. These biofilms were characterized by a lower number of cyanobacterial taxa, although the dominant taxa appeared to be very abundant and sometimes formed thick phototrophic layers. However, the diatoms in such biofilms were quite diverse, which is demonstrated by PCA (Figure 5). Chl *a* concentrations confirm high primary production in such biofilms, with the highest values measured in Omoljica and Poljane, particularly at Om T2 (48.66 µg/cm^2^) and Po T1 (65.29 µg/cm^2^) (Figure 3). These sites, with their higher temperatures (20–25 °C) and mineral content, provide a suitable environment for photosynthetic microbial activity. In contrast, the biofilms on the walls of the taps, which were occasionally splashed by thermomineral water, were less developed, probably due to the limited water contact and frequent desiccation. These epilithic biofilms were more strongly associated with the substrate and were dominated by stress-tolerant organisms, particularly coccoid Cyanobacteria (Figure 5), and, in some cases, resembled aerophytic biofilms in terms of appearance and community composition. Considering the Chl *a* concentration, lower values are observed, particularly in biofilms like those from the Bukovička Spa. Many of the biofilms examined appeared to be gelatinous or mucilaginous, meaning that they are rich in extracellular substances (EPS) produced by phototrophic organisms, especially Cyanobacteria [48]. These EPS are very useful for microorganisms in biofilms and have many functions, including the retention of water (in the case of biofilms that develop on tap walls), the accumulation of many beneficial substances for the growth and development of microorganisms, and various types of protection (from desiccation, predators, and toxic substances) [48]. The observed color variations may indicate differences in the composition of microorganisms and the dominance of certain Cyanobacteria and eucaryotic algae that thrive in mineral-rich environments with high temperatures [49].

Cyanobacteria, which are adapted to extreme environments, often dominate under these conditions, especially in hot springs [50]. The cyanobacterial community in the investigated thermomineral springs showed considerable diversity, with simple trichal forms dominating, followed by coccoid representatives. A similar trend was observed by Šaraba et al. when studying biofilms at six localities in Serbia, where *Gloeocapsa*, *Leptolynbgya* and *Phormidium* were also listed among the dominant genera in terms of diversity [24]. The dominance of filamentous Cyanobacteria and the higher diversity of representatives of the genera *Leptolynbgya* and *Phormidium* is to be expected, as these taxa are typical inhabitants of thermal environments and are highly resistant to elevated temperatures and variable mineral concentrations [40,46]. Filamentous forms, especially *Leptolyngbya boryana* and *Leptolyngbya thermalis* Anagnostidis, were detected in several thermomineral springs, reflecting their ecological importance [51]. In contrast, coccoid Cyanobacteria such as *Gloeocapsa* and *Synechococcus*, as well as representatives of *Aphanocapsa* and *Chroococcus* detected at the studied sites, showed a preference for specific locations where biofilms develop in the splash zone at the wall of taps. According to Ward et al., studies on European thermal springs have shown that Cyanobacteria such as *Synechococcus* and *Chlorogloeopsis* are common, especially at places where elevated temperatures and the availability of light promote their growth [52]. Unique taxa were observed at individual sites, indicating localized environmental niches and the importance of possible microenvironmental parameters. *Chroococcus thermalis* was found only in Vrujci (Vr T1), and *Pseudanabaena thermalis* was detected only in Omoljica (Om T1). This is consistent with findings from hot springs in Greece, where certain taxa were restricted to specific thermal conditions [46]. *Jaaginema geminatum* has been associated with thermal habitats, including hot and thermomineral springs, where it thrives under certain physico-chemical conditions. For example, thermal springs in Turkey and other regions host Cyanobacteria such as *Jaaginema* [41], which are adapted to warm, mineral-rich water, similar to Omoljica, the place where this taxon has only been recorded in this research. *Aphanothece saxicola* is a species commonly found in various environments, including rocky surfaces [28] and, occasionally, in habitats such as thermal and mineral springs. Its adaptation to mineral-rich or thermal conditions is remarkable, making it a potential inhabitant of thermal springs [53]. *A. saxicola* has only been recorded in Vrujci. As far as the studied localities are concerned, Bukovička, Vrujci, and Omoljica showed the highest cyanobacterial diversity. Table 3 shows that in the Bukovička spa, coccoid representatives dominate in the biofilms sampled mainly from the splash zone on the walls of the taps, while in the Omoljica spa, simple trichal representatives dominate, but in the biofilms that were fully submerged in water. In Vrujci, where both types of biofilms were present, the diversity of both coccoid and simple trichal forms was high. In contrast, there were fewer cyanobacterial taxa in Poljane, indicating its particular ecological conditions. Only a few taxa were also found in Ovčanska spa. It is known that in places with specific environmental parameters, there are fewer taxa adapted to the existing conditions, which is especially true for thermomineral springs [11].

A molecular study was conducted to confirm the dominant taxa, to improve our understanding of the biodiversity and ecological role of cyanobacteria in the studied thermomineral springs, and to construct a phylogenetic tree to show evolutionary links between these taxa. The detection of rare Cyanobacteria, such as *Desertifilum tharense* Dadheech and Krienitz (Ov T1 sediment) and *Wilmottia murrayi* (West and G.S.West) Strunecký, Elster, and Komárek (Bu T3), highlights the presence of unique habitats that can host highly specialized phototrophs. None of these taxa have been explicitly documented in thermomineral or hot springs, and studies confirming their occurrence in such environments are lacking [54,55]. *D. tharense* was only recorded in Romania in Europe, and *W. murray* has not been recorded at all in Europe so far. These findings align with reports of similar taxa in extreme thermal environments worldwide [42]. The filamentous genera, whose role in microbial mats is well documented [56], probably play a crucial role in primary production and biogeochemical cycling in these springs. The presence of *Elainella* sp. in Bukovička Spa–Knjaz Mihailo is significant, as species of this genus have primarily been associated with extreme environments [57]. Its detection via 16S rRNA molecular analysis expands its known ecological range. The specific environmental parameters of Bukovička Spa–Knjaz Mihailo may mimic certain aspects of the genus’s preferred habitat, such as the availability of essential nutrients like phosphorus (0.085 mg/L total phosphorus) and silicates (22.355 mg/L) and types of biofilms that develop in splash-zone, partially being moistened by thermomineral water and partially being aerophytic. This finding supports the genomic plasticity of *Elainella* sp., which has been highlighted in whole-genome studies as essential for survival in diverse ecological contexts [57]. The detection of *Geitlerinema* sp. exclusively in Poljane underscores its ecological specialization in thermomineral environments. The highest Chl *a* concentration measured in Poljane (>65 µg/cm^2^) reflects the high primary productivity of the site, likely influenced by optimal physical and chemical conditions for photosynthetic microorganisms. Molecular analysis using 16S rRNA sequencing confirmed the presence of *Geitlerinema* sp. in this hot spring. Similar studies in hot springs worldwide, such as those in Algeria and Mexico, have identified *Geitlerinema* as a key taxon in microbial mats, often associated with thermophilic conditions [58,59]. The *Geitlerinema* species has also been detected in mineral sources in Belotić and Radaljska banja in Serbia [24]. *Nodosilinea* species inhabit diverse environments, including terrestrial, aquatic, and extreme habitats such as radioactive thermal springs and saline conditions [60]. Their adaptability is attributed to robust cell wall structures and efficient photosynthetic mechanisms. *Nodosilinea* sp. was isolated and cultured from three thermomineral spas (Bukovička spa, Ovčanska spa, and Omoljica). These Cyanobacteria are characterized by simple trichal forms, often in appearance similar to *Leptolyngbya* in morphology, with thin, filamentous cells [61]. For some taxa characterized in this study, few data are available, and the shown results added to the knowledge of their distribution, which appears to be larger than previously documented. Further research in these and other extreme habitats worldwide will likely lead to a more comprehensive understanding. The cultivation and characterization of such taxa is also crucial for future research, especially in the context of biotechnological applications.

The diatom community was the most diverse group in this study. The most species-rich genera were *Nitzschia* and *Navicula*, which is consistent with reports from other European thermal springs [40]. Studies from Galicia (NW Spain) also highlight *Navicula* and *Nitzschia* as dominant genera, indicating a shared adaptability of these taxa to thermomineral conditions in different regions [45]. *Nitzschia palea*, which was recorded in all five springs, emphasizes its adaptability to the eutrophic and variable thermomineral conditions characteristic of these environments [44]. Several taxa were found in a few localities, including *Nitzschia thermaloides*, which was found in the Vrujci, Omoljica, and Ovčanska spas, indicating overlapping environmental gradients. However, many taxa were found only in one spring; *Adlafia muralis* was detected only in Poljane, where the water temperature was 17.4 °C. This species is a freshwater inhabitant of waters with high trophic level, whose heat tolerance is not well documented, according to Lange-Bertalot et al. [34]. Vrujci proved to be a hotspot of diatom diversity. A significant difference in species richness was found in Vrujci, with almost twice as many taxa occurring in the natural source (biofilms that were fully submerged) than at the tap (biofilms that were splashed by thermomineral water). A similar disparity in diatom diversity between submerged and splashed biofilms has been noted in Berchtesgaden National Park (Germany), where site-specific conditions greatly influence species richness [44]. In Vrujci, the natural spring and the tap also differ in terms of species richness and composition. The Bukovička spa had the lowest diversity, dominated by *Gomphonema parvulum* (Kützing) Kützing and *Achnanthidium exiguum* (Grunow) Czarnecki. These taxa are known to thrive in systems with fluctuating ion concentrations and organic pollution [43]. However, the lowest number of diatom taxa in the Bukovička spa is expected and confirmed by the PCA (Figure 5), considering that the phototrophic biofilms were sampled from taps walls and splashing zones. The diatom results of our study show that it is difficult to define a diatom community that is characteristic of thermomineral springs. Some of the species are widely distributed and have a broad ecological preference for many environmental factors, but there are also species with a narrow preference whose distribution depends on the site-specific conditions, microclimatic parameters, and physical and chemical parameters of the individual spring.

When considering the statistical analyses (Figure 6), it should be noted that only three parameters proved to be significant (ammonia, TN, and TOC), which is probably due to the fact that there are different samples (i.e., types of biofilms, sediment) from which the taxa are identified. The water parameters will certainly have more influence on those taxa developing in sediment or biofilms that are fully submerged than on those that form biofilms in the splash zone, which may also be influenced by other additional site-specific parameters.

## 5. Conclusions

Thermomineral springs represent places with high ecological diversity and a complexity of phototrophic biofilms. Our research emphasized the dependence of phototrophic biofilms on the unique physical and chemical properties of each spring. The results show considerable variability in water parameters such as temperature, pH, turbidity, and ion concentration, which influence the structure and composition of biofilms. Submerged biofilms, which are fully submerged, had higher Chl *a* concentrations and a greater abundance of filamentous Cyanobacteria, while biofilms in the splash zone were dominated by coccoid Cyanobacteria. The detection of rare taxa such as representatives of *Wilmottia*, *Desertifilum*, *Elainella*, *Nodosilinea* and *Geitlerinema* by molecular analysis underlines the potential of the springs to harbor highly specialized microorganisms. The diatom communities showed site-specific variations reflecting the microenvironmental conditions of the springs. Thermomineral springs in Serbia still represent “hidden gems”, underexplored places, with great potential in terms of diversity and, especially, highly unique and taxa still not explored enough in an ecological or applicable manner.

## Figures and Tables

**Table 1:** List of thermomineral springs, coordinates, and designations of sampling sites.

Locality	Site	Coordinates	Source
Bukovička Spa	Bu T1	44.3086948 N, 20.5525070 E	Topla česma tap
	Bu T2		
	Bu T3		
	Bu T4	44.3079347 N, 20.5510747 E	Knjaz Mihailo tap
	Bu M		Mud
Omoljica	Om T1	44.3079347 N, 20.5510747 E	Natural pool
	Om T2		Natural pool
	Om S		Sediment
Ovčanska Spa	Ov T1	44.76168396 N, 20.75283817 E	Natural pool
	Ov S		Sediment
Vrujci	Vr S	44.2204950 N, 20.1521023 E	Sediment
	Vr T1		Natural pool
	Vr T2	44.2203266 N, 20.1524234 E	Vrujci tap
	Vr T3		Vrujci tap
	Vr M		Mud
Poljane	Po T1	44.52263555 N, 20.2366812 E	Pipe where water barely drips

**Table 2:** Values of physical and chemical water parameters in studied thermomineral springs.

Parameters	Bukovička Spa		Omoljica	Ovčanska Spa	Vrujci	Poljane
	Bukovička Spa–Topli Izvor	Bukovička Spa–Knjaz Mihailo				
Water Temperature (°C)	17.5	8.5	22	20.3	25.1	17.4
Turbidity (NTU)	9.46	0.87	0.82	4.24	0.04	0.02
pH Value	6.86	7.63	8.1	7.59	7.45	8.19
TDS (ppt)	3.2	0.1	1.2	17.6	0.382	0.9
EC (ms)	4.6	0.2	1.8	/	0.5	1.3
O_2_ (mg/L)	2.9	10.94	2.46	9.38	6.6	2.1
O_2_ (% saturation)	16.6	92.5	27.5	91	62.2	17.6
Conductivity at 20 °C (µS/cm)	4220	233	1744	2550	461	1187
Ammonia, NH_4_-N (mg/L)	0.05	0.06	2.52	19	<0.05	7.89
Nitrites, NO_2_-N (mg/L)	<0.005	<0.005	<0.005	<0.005	<0.005	<0.005
Nitrates, NO_3_-N (mg/L)	<0.5	0.9	<0.5	20	2.01	<0.5
Chlorides, Cl^−^ (mg/L)	27.6	7.9	31.7	995	3.61	25.44
Sulfates, SO_4_^2−^ (mg/L)	1.2	42.2	9.8	<0.5	10	3.25
Fluorides, F (mg/L)	5	0.1	1.3	<0.05	0.75	3.19
Bromides, Br (mg/L)	<0.5	<0.5	<0.5	<0.5	<0.5	<0.5
Silicates, SiO_2_ (mg/L)	81.59	7.67	17.97	28	13.179	22.355
Orthophosphates, PO_4_-P (mg/L)	0.019	<0.01	0.087	0.011	0.054	0.043
Total Phosphorus (mg/L)	0.044	0.008	0.114	0.051	0.154	0.085
Calcium, Ca^2+^ (mg/L)	86.1	36.8	4	44	75.3	6
Magnesium, Mg^2+^ (mg/L)	16.3	7.8	10.7	86.5	17.5	6.8
Total Hardness (dH)	15.7	6.9	3	26	15	2.4
Alkalinity (n/10HCl)	601	22	197	104	55	143
Bicarbonates (mg/L)	3665	134	1202	634	336	872
Total Organic Carbon (TOC) (mg/L)	2.94	1.41	5.64	1.61	2.33	3.47
Total Nitrogen (TN) (mg/L)	<0.5	<0.5	2	19	0.8	3.36

**Table 3:** Identified cyanobacterial and algal taxa. Taxa marked with an asterisk (*) were identified from cultures using molecular methods.

Identified Phototrophicmicroorganisms	Bukovička Spa	Omoljica	Ovčanska Spa	Vrujci	Poljane
Cyanobacteria					
*Anagnostidinema amphibium* (Gomont) Strunecký, Bohunická, J.R.Johansen and Komárek					+
*Aphanocapsa fusco-lutea* Hansgirg	+	+		+	
*Aphanocapsa* Nägeli sp.				+	
*Aphanothece saxicola* Nägeli				+	
*Arthrospira* Stizenberger ex Gomont sp.				+	
*Chroococcidiopsis* Geitler sp.	+				
*Chroococcus thermalis* (Meneghini) Nägeli				+	
*Chroococcus varius* A.Braun	+			+	
*Cyanosarcina* Kovácik sp.	+				
*Desertifilum tharense* * Dadheech and Krienitz			+		
*Elainella* Jahodárová, Dvorák and Hasler sp. *	+				
*Geitlerinema* (Anagnostidis and Komárek) Anagnostidis sp. *					+
*Gloeocapsa atrata* Kützing	+			+	
*Gloeocapsa fusco-lutea* Nägeli ex Kützing/Ercegović	+				
*Gloeocapsa punctata* Nägeli	+			+	
*Gloeocapsa violacea* Kützing	+			+	
*Jaaginema geminatum* (Schwabe ex Gomont) Anagnostidis and Komárek		+			
*Kamptonema jasorvense* (Vouk) Strunecký, Komárek and J.Smarda		+			
*Leptolyngbya boryana* * (Gomont) Anagnostidis and Komárek	+	+		+	
*Leptolyngbya foveolarum* (Gomont) Anagnostidis and Komárek	+		+	+	
*Leptolyngbya perforans* (Geitler) Anagnostidis and Komárek				+	
*Leptolyngbya* Anagnostidis and Komárek sp. *		+		+	
*Leptolyngbya thermalis* Anagnostidis		+			
*Nodosilinea* R.B.Perkerson and D.A.Casamatta sp. *	+	+	+		
*Oscillatoria* cf. *nitida* Vaucher ex Gomont		+			
*Oscillatoria* Vaucher ex Gomont sp. *				+	
*Oscillatoria subbrevis* Schmidle		+			
*Phormidium* cf. *terebriforme* Kützing ex Gomont					+
*Phormidium corium* Gomont				+	
*Phormidium kuetzingianum* (Kirchner ex Hansgirg) Anagnostidis and Komárek	+				
*Phormidium laetevirens* (P.Crouan and H.Crouan ex Gomont) Anagnostidis and Komárek		+	+		
*Phormidium* Kützing ex Gomont sp.1	+				
*Phormidium* Kützing ex Gomont sp.2				+	
*Phormidium* Kützing ex Gomont sp.3				+	
*Phormidium* Kützing ex Gomont sp.4		+			
*Phormidium* Kützing ex Gomont sp.5		+			
*Phormidium tergestinum* (Rabenhorst ex Gomont) Anagnostidis and Komárek				+	
*Porphyrosiphon notarisii* Kützing ex Gomont				+	
*Pseudanabaena minima* (G.S.An) Anagnostidis		+			
*Pseudanabaena thermalis* Anagnostidis		+			
*Synechococcus bigranulatus* Skuja	+	+			
*Synechococcus elongatus* (Nägeli) Nägeli	+				
*Synechococcus* Nägeli sp.	+			+	
*Synechocystis* Sauvageau sp.			+		
*Wilmottia murrayi* * (West & G.S.West) Strunecký, Elster, and Komárek	+				
*Wilmottia* O.Strunecky, J.Elster, and J.Komárek sp. *				+	
*Wolskyella* cf. *floridana* Claus		+		+	
Bacillariophyta					
*Achnanthes coarctata* (Brébisson ex W.Smith) Grunow			+		
*Achnanthidium exiguum* (Grunow) Czarnecki	+	+	+	+	+
*Achnanthidium gracillimum* (F.Meister) Lange-Bertalot				+	
*Achnanthidium microcephalum* Kützing			+	+	+
*Achnanthidium straubianum* (Lange-Bertalot) Lange-Bertalot				+	
*Adlafia bryophila* (J.B.Petersen) Lange-Bertalot				+	
*Adlafia muralis* (Grunow) Monnier and Ector					+
*Amphora pediculus* (Kützing) Grunow				+	+
*Anomoeoneis* cf. *capitata* E.Pfitzer		+			
*Anomoeoneis sphaerophora* Pfitzer			+		
*Brachysira vitrea* (Grunow) R.Ross				+	
*Caloneis fontinalis* (Grunow) A.Cleve				+	
*Caloneis silicula* (Ehrenberg) Cleve				+	
*Caloneis* Cleve sp.				+	
*Cocconeis euglypta* Ehrenberg				+	
*Cocconeis euglyptoides* (Geitler) Lange-Bertalot				+	
*Craticula ambigua* (Ehrenberg) D.G.Mann		+		+	
*Craticula* Grunow sp.		+			
*Cymbella affinis* Kützing				+	
*Denticula kuetzingii* Grunow				+	
*Encyonopsis minuta* Krammer and E.Reichardt				+	
*Gomphonema acidoclinatum* Lange-Bertalot and E.Reichardt		+			
*Gomphonema cymbelliclinum* E.Reichardt and Lange-Bertalot				+	
*Gomphonema parvulum* (Kützing) Kützing	+	+	+	+	+
*Gomphonema pumilum var rigidum* E.Reichardt and Lange-Bertalot				+	
*Gomphonema* Ehrenberg sp.1				+	
*Gomphonema* Ehrenberg sp.2				+	
*Grunowia solgensis* (A.Cleve) Aboal				+	
*Halamphora coffeiformis* (C.Agardh) Mereschkowsky			+		
*Halamphora* (Cleve) Mereschkowsky sp.				+	
*Hantzschia abundans* Lange-Bertalot		+			
*Hantzschia amphyoxis* (Ehrenberg) Grunow				+	
*Hantzschia calcifuga* E.Reichardt and Lange-Bertalot		+			
*Hantzschia* Grunow sp.			+		
*Humidophila contenta* (Grunow) Lowe, Kociolek, Johansen, Van de Vijver, Lange-Bertalot and Kopalová				+	
*Humidophila perpusilla* (Grunow) R.L.Lowe, Kociolek, J.R.Johansen, Van de Vijver, Lange-Bertalot and Kopalová			+		
*Lemnicola hungarica* (Grunow) Round and Basson			+		
*Luticola mutica* (Kützing) D.G.Mann			+		
*Melosira varians* C.Agardh		+	+		
*Navicula cincta* Pantocsek		+	+	+	
*Navicula cryptocephala* Kützing				+	
*Navicula cryptotenella* Lange-Bertalot				+	
*Navicula reichardtiana* Lange-Bertalot				+	
*Navicula rostellata* Kützing				+	
*Navicula salinarum* Grunow			+		
*Navicula* Bory sp.1	+		+	+	+
*Navicula* Bory sp.2				+	
*Navicula vandamii* Schoeman and R.E.M.Archibald				+	
*Neidium dubium* (Ehenberg) Cleve				+	
*Nitzschia acicularis* (Kützing) W.Smith				+	
*Nitzschia amphibia* Grunow				+	
*Nitzschia communis* Rabenhorst		+			+
*Nitzschia dissipata* (Kützing) Rabenhorst		+	+		
*Nitzschia fonticola* (Grunow) Grunow		+			
*Nitzschia inconspicua* Grunow				+	
*Nitzschia liebetruthii* Rabenhorst			+	+	
*Nitzschia linearis* W.Smith				+	
*Nitzschia palea* (Kützing) W.Smith	+	+	+	+	+
*Nitzschia* Hassall sp.1		+			
*Nitzschia* Hassall sp.2		+			
*Nitzschia thermaloides* Hustedt		+	+	+	
*Nitzschia umbonata* (Ehrenberg) Lange-Bertalot					+
*Pinnularia* Ehrenberg sp.				+	
*Placoneis placentula* Heinzerling				+	
*Planothidium dubium* (Grunow) Round and Bukhtiyarova				+	
*Planothidium frequentissimum* (Lange-Bertalot) Lange-Bertalot				+	+
*Planothidium reichardtii* Lange-Bertalot and Werum				+	
*Platessa* Lange-Bertalot sp.				+	
*Pseudostaurosira brevistriata* (Grunow) D.M.Williams and Round				+	
*Reimeria uniseriata* Sala, Guerrero and Ferrario				+	
*Sellaphora bacillum* (Ehrenberg) D.G.Mann				+	
*Sellaphora* complex *pupula* Mereschowsky				+	
*Sellaphora laevissima* (Kützing) D.G.Mann				+	
*Sellaphora* Mereschowsky sp.	+			+	
*Sellaphora stroemii* (Hustedt) H.Kobayasi				+	
*Sellaphora ventraloides* (Hustedt) Falasco and Ector				+	
*Stephanocyclus meneghiniana* (Kützing) Kulikovskiy, Genkal and Kociolek			+		
*Staurosira* Ehrenberg sp.				+	
*Surirella ovalis* Brébisson		+			+
*Surirella splendida* (Ehrenberg) Ehrenberg				+	
*Ulnaria ulna* (Nitzsch) Compère				+	
Chlorophyta					
*Desmococcus olivaceus* (Persoon ex Acharius) J.R.Laundon				+	
*Scenedesmus* Meyen sp.		+			

## Data Availability

The original contributions presented in this study are included in the article. Further inquiries can be directed to the corresponding author.
